# Mechanisms and microbial influences on CTLA-4 and PD-1-based immunotherapy in the treatment of cancer: a narrative review

**DOI:** 10.1186/s13099-020-00381-6

**Published:** 2020-09-10

**Authors:** Peter L. Miller, Tiffany L. Carson

**Affiliations:** 1 University of Alabama at Birmingham School of Medicine, Birmingham, AL USA; 2 Department of Medicine, Division of Preventive Medicine, University of Alabama Birmingham School of Medicine, 1717 11th Ave S, Birmingham, AL 35205 USA

**Keywords:** Gut microbiota, Cancer, Immunotherapy, Oncomicrobiotics

## Abstract

**Background:**

The relationship between gastrointestinal (GI) bacteria and the response to anti-CTLA-4 and anti-PD-1 immunotherapy in the treatment of cancer can potentially be enhanced to allow patients to maximally respond to these treatments. Insight into the complex interaction between gut microbiota and the human adaptive immune system will help guide future immunotherapeutic cancer treatments to allow a more robust clinical response and fewer adverse effects in patients requiring these drugs. This review highlights these interactions as well as the potential for the creation of “oncomicrobiotics” that would selectively tailor one’s GI bacteria to maximally respond to anti-CTLA-4 and anti-PD-1 treatments will fewer adverse effects.

**Main body:**

CTLA-4 is an antigen on the surface of T cells which, upon stimulation, leads to inhibition of activated T cells to terminate the immune response. However, many types of tumor cells can upregulate CTLA-4 in the tumor microenvironment, allowing these cells to evade targeting and destruction by the body’s immune system by prematurely inhibiting T cells. Increased representation of *Bacteroides fragilis*, *Burkholderia cepacia* and the *Faecalibacterium genus* in the GI tract of patients receiving CTLA-4-based immunotherapy led to a stronger therapeutic effect while minimizing adverse side effects such as colitis. In addition, by introducing bacteria involved in vitamin B and polyamine transport to the GI tracts of patients treated with anti-CTLA-4 drugs led to increased resistance to colitis while maintaining therapeutic efficacy. PD-1 is another molecule upregulated in many tumor microenvironments which acts in a similar manner to CTLA-4 to tone down the anti-neoplastic actions of T cells. Antibodies to PD-1 have shown promise to help allow the body’s natural immune response to appropriately target and destroy tumor cells. The presence of *Bifidobacterium breve* and *longum*, *Akkermansia muciniphila* and *Faecalibacterium prausnitzii* in the GI tracts of cancer patients has the potential to create a more robust immune response to anti-PD-1 drugs and prolonged survival. The development of “oncomicrobiotics” has the potential to help tailor one’s gut microbiota to allow patients to maximally respond to immunotherapy without sacrificing increases in toxicity. These oncomicrobiotics may possibly include antibiotics, probiotics, postbiotics and/or prebiotics. However, many challenges lie ahead in the creation of oncomicrobiotics.

**Conclusion:**

The creation of oncomicrobiotics may allow many patients receiving anti-CTLA-4 and PD-1 immunotherapy to experience prolonged survival and a better quality of life.

## Background

The influence of gut microbiota on immunotherapeutic cancer treatments is gaining popularity in recent literature. This literature review investigates the role of gut bacteria in anti-CTLA-4 and anti-PD-1 immunotherapy and possible “oncomicrobiotics” that can potentially lead to a more robust response to these treatments. Ideal oncomicrobiotics would help tailor one’s gut microbiota to a desired composition for maximum response to immunotherapy with less adverse effects. Many challenges plague the creation of such oncomicrobiotics, which will be discussed in more detail in later sections.

The commensal relationship between humans and bacteria is very complex and continues to evolve. The majority of microbes that inhabit the human body are bacteria [1]. The vast majority (99%) of these commensal bacteria are present in the human gastrointestinal (GI) tract, mostly in the distal colon [2]. The average human colon houses trillions of bacteria [2, 3]. Collectively the bacteria that inhabit the human body make up what is referred to as the microbiota, and body sites (e.g., gut, skin, oral cavity) have niche-specific microbiota.

Recently much attention has been dedicated to investigating the relationship between humans and the bacteria inhabiting their GI tracts and how these bacteria influence diseases and disease treatments [1, 2, 4, 5]. The symbiosis between humans and bacteria is a mutualistic relationship [6]. Gut bacteria breakdown indigestible compounds and occupy niches and space in the human GI tract that may be otherwise filled with more pathogenic bacteria. On the contrary, humans provide a protected environment and abundant nutrients to gut microbes [7].

There is a significant difference in the composition of GI bacteria between different individuals and many factors influence the composition of one’s gut microbiota [8]. These factors include but are not limited to: mode of delivery at birth (vaginal versus Cesarean section), ingestion of breast milk versus formula during infancy, diet, medications and exposure to environmental agents [8]. The variation in the composition of gut bacteria across populations makes it increasingly difficult to determine how these bacteria influence one’s health. In addition, many of these bacteria are unable to be cultured in the laboratory, which poses quite the challenge when studying them [9].

Symbiosis is the intimate relationship between two organisms living together in close proximity. Symbiosis can occur as commensalism in which one party benefits while the other is unaffected, mutualism in which both parties benefit or parasitism in which one party benefits at the expense of the other [6]. “Dysbiosis” is the term used to describe an altered host-gut microbiota relationship. Dysbiosis has been linked to many diseases including type 2 diabetes, inflammatory bowel disease, autoimmune diseases and neurological diseases [10]. Dysbiosis can not only lead to disease, it can also affect treatments for many diseases. Gut microbes have been shown to influence both innate and adaptive immunity in many ways, but the mechanisms underlying the specific processes are less known. The ongoing evolution of the human immune system makes it increasingly difficult to delineate how gut microbes mediate its effects [10].

Interestingly, the composition of one’s gut bacteria affects the efficacy and toxicity of immunotherapeutic treatments for certain types of cancer. Eighteen percent of cancers worldwide are attributable to infectious agents, including human papillomavirus (HPV) in cervical cancer, hepatitis C virus in hepatocellular carcinoma and *H. pylori* in gastric cancers [2, 11, 12]. Some viruses, such as HPV, can cause cancer via distinct genetic mechanisms while other microbes, like *H. pylori*, lead to local inflammation and epithelial disruption [11, 12]. In the past, much research investigating the role of gut microbes in the development of cancer has focused on colorectal cancer. It is now clear that gut microbiota are able to influence carcinogenesis both locally and systemically [13].

While antibiotic-treated germ-free mice (GF mice), which lack gut bacteria, seem to show reduced risk for some types of cancer, the presence of specific gut bacteria are required for the efficacy of some immunotherapeutic treatments. This suggests that gut microbes may have anti-tumor effects as well as carcinogenic potential [2]. Further investigation into how GI bacteria influence cancer treatments will improve the efficacy of these treatments.

Although the composition of one’s gut microbiota may influence chemotherapy, radiation and other cancer treatments, this review will focus on how GI bacteria influence immunotherapy. Immunotherapy is the use of the body’s own immune system to attack tumor cells, mainly via activation of T cells and downstream cytotoxic effects. Many tumor cells are unrecognizable by T cells and/or have the ability to inactivate T cells by various means. This allows tumor cells to go undetected by the immune system and proliferate uncontrollably.

The activation of T cells to target and destroy cells (e.g. tumor cells) requires 2 signals. T cells must first recognize an antigen in the context of a major histocompatibility complex (MHC) molecule on an antigen presenting cell (APC). The second signal relies on the co-stimulation between the B7 surface molecule of the APC and the CD28 surface molecule on the T cell [14]. To terminate this co-stimulation, T cells express cytotoxic T lymphocyte antigen 4 (CTLA-4) on their surface. CTLA-4 is a co-inhibitory ligand that binds B7 with a higher affinity than CD28 and this interaction inactivates the primed T cell. The CTLA-4 interaction allows termination of the immune response. In addition, T cells express programmed death 1 (PD-1) that binds to PD-L1 or PD-L2 of other cells (e.g. tumor cells) to terminate the T cell response similarly to CTLA-4. PD-L1 is expressed on many types of tumor cells including lung cancer, melanoma, breast cancer, hepatocellular carcinoma, gastric cancer and pancreatic cancer [15]. During early development, T cells undergo negative selection in the thymus which prevents them from targeting self-antigens. Tumor cells express self-antigens and thus are not adequately targeted for destruction by T cells. Leach et al. demonstrated that blocking the CTLA-4 receptor led to an increased anti-tumor immune response, suggesting that tumor cells are capable of upregulating CTLA-4 in the tumor microenvironment to avoid detection by the immune system [16].

Antibodies against CTLA-4 and PD-1/PD-L1 have shown to improve overall survival in many patients with different types of cancer including melanoma, non-small cell lung cancer (NSCLC), renal cell carcinoma, hepatocellular carcinoma, head and neck squamous cell carcinoma and bladder cancer [15, 17]. The goal of these treatments is to restoret the anti-tumor responses of T cells [18]. By blocking the inhibitory pathways of T cells, tumor cells are more susceptible to being targeted and destroyed by T cells. These anti-CTLA-4 and anti-PD-1/PD-L1 antibodies are referred to as immune checkpoint blockades (ICBs).

Although ICBs have been shown to improve overall survival in some cancer patients, only certain tumors express PD-L1. These tumors include squamous carcinoma of head and neck, melanoma and various tumors of the brain, thymus, thyroid, esophagus, liver and pancreas [15]. However, most early research on these treatments has focused on malignant melanoma and only a subset of patients show clinical responses, often with variable responses and sustainability [19–21]. In 2007, Paulos et al. demonstrated that mice treated with antibiotics showed a diminished immune response to melanoma cells due to the abolished interaction of gut microbes with the TLR4 receptor [21]. These treatments seem to be influenced by gut microbiota although the exact mechanism is still unclear. Even though cancer treatments using these ICBs have shown promise, they often result in immune-related adverse events (IRAEs) which resemble autoimmune diseases due to the breakdown of self-tolerance. While autoimmunity results from the breakdown of self-tolerance, cancer can develop due to increased self-tolerance [4]. Thus, ICB therapy acts to decrease self-tolerance and may lead IRAEs.

Since ICB therapy is unsuccessful in some patients and commonly leads to IRAEs, treatment must be balanced to provide maximum efficacy while limiting toxicity. Although many factors dictate the efficacy and toxicity of immunotherapy, it is possible that a specific composition of GI bacteria would allow patients to maximally respond to ICB therapy with fewer IRAEs [22]. The creation of “oncomicrobiotics”, medications that selectively alter one’s GI bacteria, would help tailor the composition of the microbiome to maximally respond to ICB therapy with fewer IRAEs. Oncomicrobiotics are discussed in detail at the end of this review.

## CTLA-4

### CTLA-4 as a target for immunotherapeutic cancer treatments

In 1996, Leach et al. demonstrated that blocking CTLA-4 lead to enhanced targeting of tumor cells by the body’s immune system and revealed that tumor cells have the ability to up-regulate CTLA-4 expression [16]. Thus, the increased concentration of CTLA-4 in the tumor microenvironment results in decreased activation of T cells and therefore decreased targeting and destruction of tumor cells by the body’s immune system.

Regulatory T cells (Tregs) are responsible for inhibiting cytotoxic T lymphocytes (CTLs) to prevent overactivation of the immune system and subsequent autoimmunity. Tregs highly express CTLA-4 on their surface for rapid inhibition of CTLs to keep them from exerting their cytotoxic effects [23, 24]. The monoclonal antibody ipilimumab binds CTLA-4 and leads to decreased inhibition of the anti-tumor effects of CTLs [4]. Ipilimumab has shown to be effective in patients with melanoma, lymphoma, renal cell carcinoma, urothelial carcinoma, ovarian cancer and non-small cell lung cancer^,^ [5, 22]. However, only certain subsets of patients respond to ipilimumab.

In one study of the 64 melanoma patients receiving anti-CTLA-4 therapy, only 11 patients showed long-term benefit and 14 patients showed little to no benefit [25]. Also, up to 1/3 of patients on CTLA-4 therapy develop colitis [13, 26]. Anti-CTLA-4 antibodies cause increased intraepithelial lymphocyte-mediated (IEL) apoptosis of intestinal epithelial cells (IECs) [4, 5]. Many times, CTLA-4 blockade therapy must be discontinued due to IRAEs experienced by the patient. More research needs to be dedicated to investigating which patients should receive this therapy.

### Influences of gut microbiota on CTLA-4 therapy

Although the complete mechanism of the anti-tumor effects of ipilimumab are still under debate, it is clear that gut microbiota influence the CTLA-4 blockade because GF mice fail to respond to this ICB [22]. Administration of anti-CTLA-4 antibodies promotes dysbiosis by increasing the representation of Clostridiales bacteria and decreasing Bacteroidales and Burkholderiales bacteria in the gut [1, 4, 5]. However, the frequency of *Bacteroides fragilis* (*B. fragilis*) in the gut remains somewhat constant after treatment with anti-CTLA-4 antibodies. *B. fragilis* acts in the human GI tract to help prevent and cure inflammation [27]. It is possible that *B. fragilis* plays an immunoregulatory role in the CTLA-4 pathway by production of polysaccharide A, which is hypothesized to upregulate the production of IL-10 to decrease inflammation [28]. In fact, increased representation of *B. fragilis* in feces correlated to decreased tumor size in patients treated with ipilimumab. Also, fecal transplant studies from humans to GF mice show that treatment of melanoma with the CTLA-4 blockade favors outgrowth of *B. fragilis* in the colon [5]. *B. fragilis* seems to greatly influence the efficacy of ipilimumab, but the mechanism by which this occurs is not well defined.

**Fig. 1:**
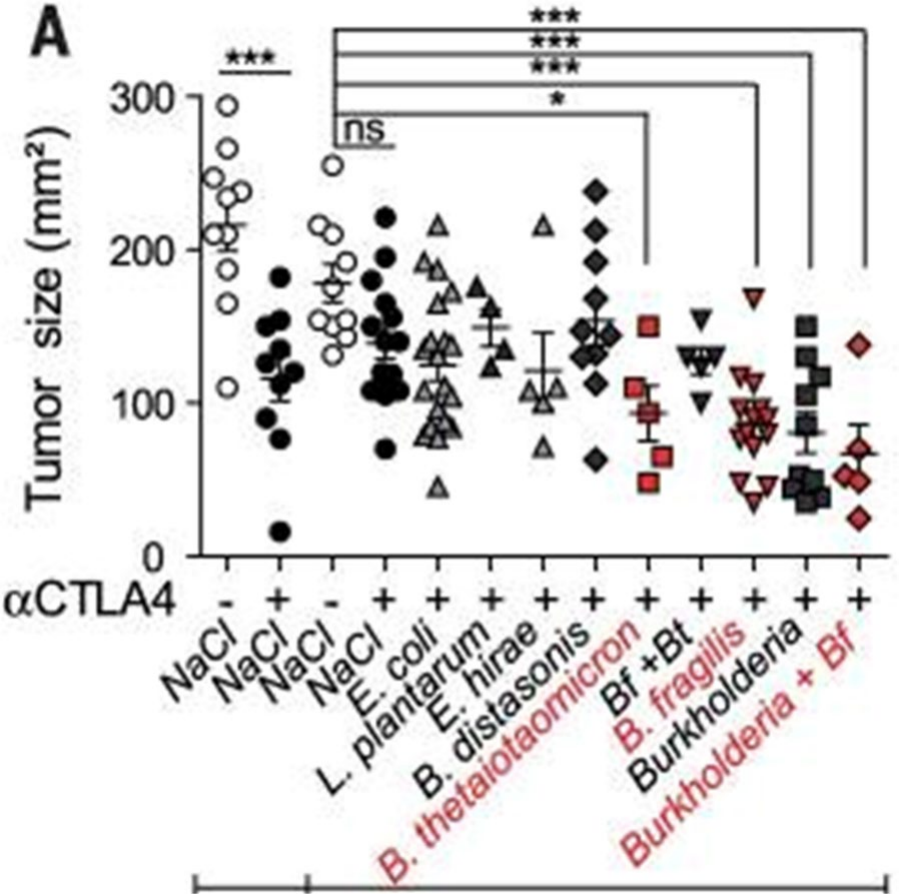
Tumor size after oral feeding of bacteria to GF mice treated with anti-CTLA-1 therapy. Oral supplementation of *B. fragilis*, *B. theraiotaomicron* or Burkholderia cepacia + *B. fragilis* to GF mice leads to a more robust response to anti-CTLA-4 therapy and subsequent decrease in tumor size [14]. Figure taken from Vetizou et al.

**Fig. 2:**
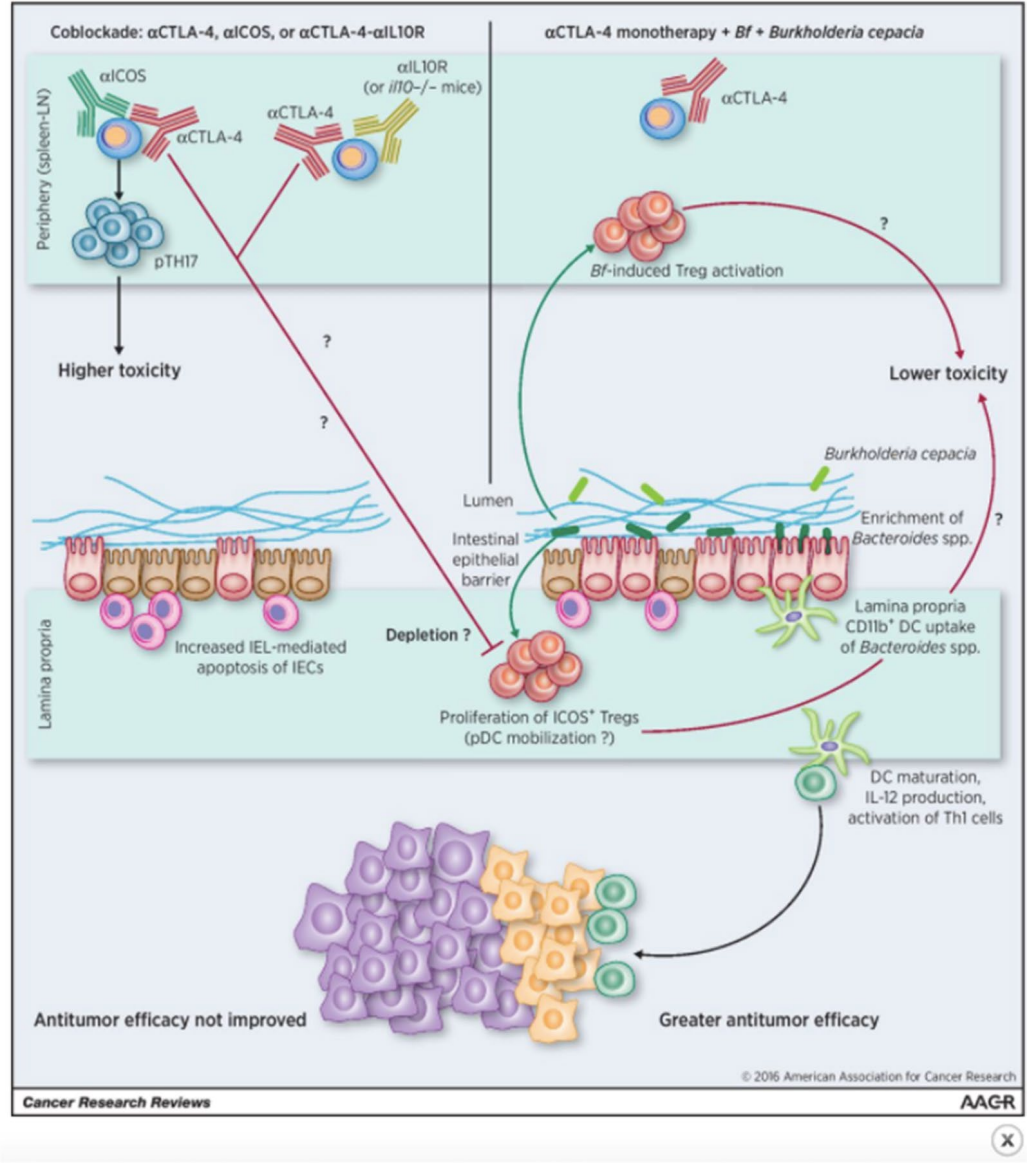
Effects of *B. fragilis* + *B. cepacia* on antitumor response to CTLA-4 therapy. Recolonization of GF mice with *B. fragilis* and *B. cepacia* leads to greater antitumor response to anti-CTLA-4 therapy via DC maturation, IL-12 production and activation of Th1 cells [11, 14]. Figure taken from Pitt et al.

Oral administration of *B. fragilis*, *Bacteroides thetaiotaomicron* (*B. thetaiotaomicron*), or a combination of *B. fragilis* and *Burkholderia cepacia* (*B. cepacia*) to GF mice allows these mice to elicit the anti-tumor immune response seen in mice with normal microbiota, leading to decreased tumor size (Fig. 1) [5]. The restored response is likely due to the ability of these bacteria to induce dendritic cell (DC) maturation and subsequent IL-12 production by DCs present in the lamina propria of the GI tract. DCs are APCs that process and present antigens to T cells for destruction. It is likely that the CD11b surface molecule is common to the DCs involved in this response [14]. The production of IL-12 by DCs stimulates T helper cells (Th1 cells) and prime them to help carry out the anti-tumor immune response (Fig. 2) [1, 4, 5]. Furthermore, adoptive transfer of *B. fragilis*-specific T cells into GF mice also restores the therapeutic efficacy of the CTLA-4 blockade [1]. This suggests that microbiota-dependent activation of T cells is required for response to anti-CTLA-4 antibodies.

Interestingly, the intestinal damage seen in mice that were previously germ-free but were recolonized with bacteria seemed to be milder following anti-CTLA-4 therapy compared to the intestinal lesions seen in mice with normal gut bacteria. Recolonization of gut microbiota via administration of *B. fragilis* combined with *B. cepacia* resulted in decreased intestinal damage and colitis while maintaining therapeutic efficacy [4, 5]. This suggests that these bacteria also help prevent IRAEs like colitis. Also, among patients with melanoma treated with ipilimumab, increased representation of the Bacteroidetes phylum of bacteria as well as bacteria involved in vitamin B and polyamine transport correlated to increased resistance to colitis [1]. Although the mechanism is unclear, this suggests decreased vitamin B and polyamine transport predisposes to immunotherapy-induced colitis.

In addition, Chaput et al. state that patients with melanoma that had microbiomes enriched in the Faecalibacterium genus showed longer progression-free survival as well as overall survival when treated with anti-CTLA-4 therapy. The effects seen in these patients were hypothesized to be due to decreased Treg cells in the tumor microenvironment [29]. However, Chaput el al. claims that the abundance of the Faecalibacterium genus in the gut led to an increased incidence of colitis [29]. Thus, this genus of bacteria may lead to a more robust response to ipilimumab at the cost of increased IRAEs. The effects of different bacterial species in the gut on the response to CTLA-4 therapy is summarized in Table 1 below.

**Table 1:** Effects of different bacterial species in the gut on response to CTLA-4 therapy

Bacterial species in gut	Effect on response to CTLA-4 therapy
*Bacteroides fragilis*	Increased representation in feces correlates to decrease in tumor size in melanoma-bearing mice, possibly via polysaccharide A and IL-10 production. Oral administration alone or in combination with *B. cepacia* to GF mice leads to decreased tumor size via DC maturation and IL-12 production
*Bacteroides fragilis* + *Burkholderia cepacia*	Oral administration in combination with *B. fragilis* to GF mice leads to decreased tumor size via DC maturation and IL-12 production
*Bacteroides thetaiotaomicron*	Oral administration alone to GF mice leads to decreased tumor size via DC maturation and IL-12 production
*Faecalibacterium genus*	Increased representation in the gut leads to longer progression-free and overall survival in melanoma patients due to decreased Tregs in tumor microenvironment

Certain species of bacteria are clearly needed to maximally respond to ipilimumab. Vancomycin antibiotics have been shown to decrease the abundance of gram-positive bacteria in the gut while maintaining gram-negative species such as Bacteriodales and Burkholderiales [1]. The use of vancomycin prior to anti-CTLA-4 therapy shows promise for future cancer treatments [22]. However, the use of antibiotics to selectively alter one’s microbiome faces many obstacles, including creating the opportunity of unwanted bacteria to proliferate in the human colon and killing bacteria needed for biotransformation of drugs. The challenge ahead lies in the development of “oncomicrobiotics” that specifically tailor one’s gut microbiota to maximally respond to cancer treatments.

## PD-1/PD-L1

### PD-1/PD-L1 as a target for immunotherapeutic cancer treatments

PD-1 is an inhibitory receptor present on T cells that has 2 ligands, PD-L1 and PD-L2. PD-L1 is the primary ligand and is commonly expressed by many cells in the tumor microenvironment, including tumor cells themselves and immune cells that infiltrate the tumor microenvironment [4, 30]. Similar to CTLA-4, PD-1 is expressed by activated T cells in an effort to terminate their effects. However, the expression of PD-1 is usually associated with prolonged exposure to antigens [31].

Several different antibodies have been developed that abolish the interaction between PD-1 on T cells and PD-L1 on tumor cells. These antibodies include nivolumab, pembrolizumab and lambrolizumab [4, 31]. This review will not differentiate between antibodies against PD-1 compared to antibodies against PD-L1, as the blockade results in the same effect.

These antibodies have shown objective responses in patients with melanoma, renal cell carcinoma, non-small cell lung cancer, bladder cancer, ovarian cancer and colon cancer. The most common types of cancer treated with anti-PD-1 therapy are advanced melanoma and non-small cell lung cancer [18, 22]. Depending on the study in question, the objective response rate to anti-PD-1 antibodies ranges from approximately 20–45% [18, 32, 33]. Overall progression-free survival of patients treated with these antibodies can be as high as 80% [18]. Compared to ipilimumab, melanoma patients receiving anti-PD-1 antibodies saw an increase in progression-free survival with less severe IRAEs [19]. Of the patients responding to the PD-1 blockade, about 2/3 of patients experienced clinical responses that lasted more than one year [32]. Thus, anti-PD-1 antibodies seem to show more promise than ipilimumab monotherapy alone.

Anti-CTLA-4 and anti-PD-1 antibodies both target inhibitory molecules on T cells and are used to treat similar types of cancer. However, the mechanisms by which these drugs achieve their desired effects are not the same. While the CTLA-4 blockade interferes with costimulation needed to reactivate T cells, the PD-1 blockade interferes with signaling from the TCR complex present on T cells [18, 30]. The TCR complex is a very intricate set of proteins that interact with antigens presented by APCs as well as the MHC molecule expressed on APCs. This interaction leads to a downstream signaling cascade that allows T cells to carry out their functions. Any alteration in the TCR signaling cascade can lead to disrupted T cell function [34]. The PD-1 blockade alters downstream signaling from the TCR and disrupts cellular signals involved in inhibition of T cells [18, 30]. This allows T cells to remain activated to be able to target and destroy tumor cells. Therefore, instead of reactivating T cells like the anti-CTLA-4 mechanism, the anti-PD-1 mechanism acts to keep T cells in their active state. The distinct mechanisms unique to both types of therapy indicates that combination therapy involving ipilimumab plus anti-PD-1 antibodies would be able to achieve maximum anti-tumor effects.

### Effects of gut microbiota on anti-PD-1 therapy efficacy

The PD-1/PD-L1 pathway differs from the CTLA-4 blockade by not having an absolute requirement for gut microbiota. Although no specific species of bacteria have been identified to be required for PD-1 blockade efficacy, the presence of bacteria of the genus Bifidobacterium, namely *B. breve* and *B. longum*, is strongly associated with an appropriate response to anti-PD-1 therapy [35]. In addition, high concentrations of *Akkermansia muciniphila* (*A. mucinophilia*) and *Faecalibacterium prausnitzii* (*F. prausnitzii*) in the gut have been associated with favorable responses to PD-1 therapy [17].

**Fig. 3:**
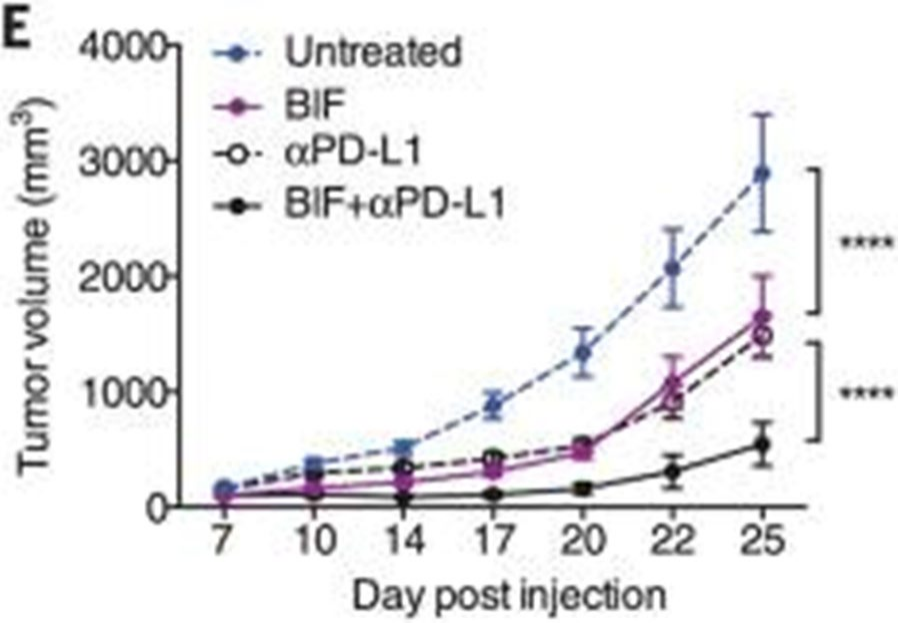
Effects of Bifidobacterium +/- PD-1 therapy on tumor size in mice. Oral administration of Bifidobacterium (BIF) improved tumor progression to the same degree as the PD-1 blockade in tumor-bearing mice. Mice treated with BIF + anti-PD-1 therapy resulted in almost complete arrest of tumor growth in this figure [22]. Figure taken from Sivan et al.

Interestingly, oral administration of *B. breve* and *B. longum* alone improved tumor progression to the same degree as the PD-1 blockade in melanoma-bearing mice [35]. Combination therapy using oral *B. breve* and *B. longum* in addition to anti-PD-1 antibodies resulted in almost complete arrest of tumor growth (Fig. 3) [35]. This observation suggests that the presence of Bifidobacterium in GI tracts of patients treated anti-PD-1 antibodies helps stimulate the immune system to adequately target tumor cells.

*Akkermansia muciniphila* is an anaerobic bacteria involved in mucous catabolism and is associated with healthy individuals without disease [17]. Routy et al. demonstrated that patients with NSCLC or renal cell carcinoma who responded to anti-PD-1 therapy had higher levels of *A. mucinophilia* represented in their microbiome compared to non-responders [17]. Similarly, Matson et al. demonstrated the same trend in patients with melanoma who responded to anti-PD-1 therapy [36]. This robust response to PD-1 therapy is thought to be due to increased production of memory T cells responsible for IFN-gamma production and subsequent tumor cell destruction [17]. Thus, abundance of *A. mucinophilia* in the gut seems to be desired in patients receiving anti-PD-1 therapy.

**Fig. 4:**
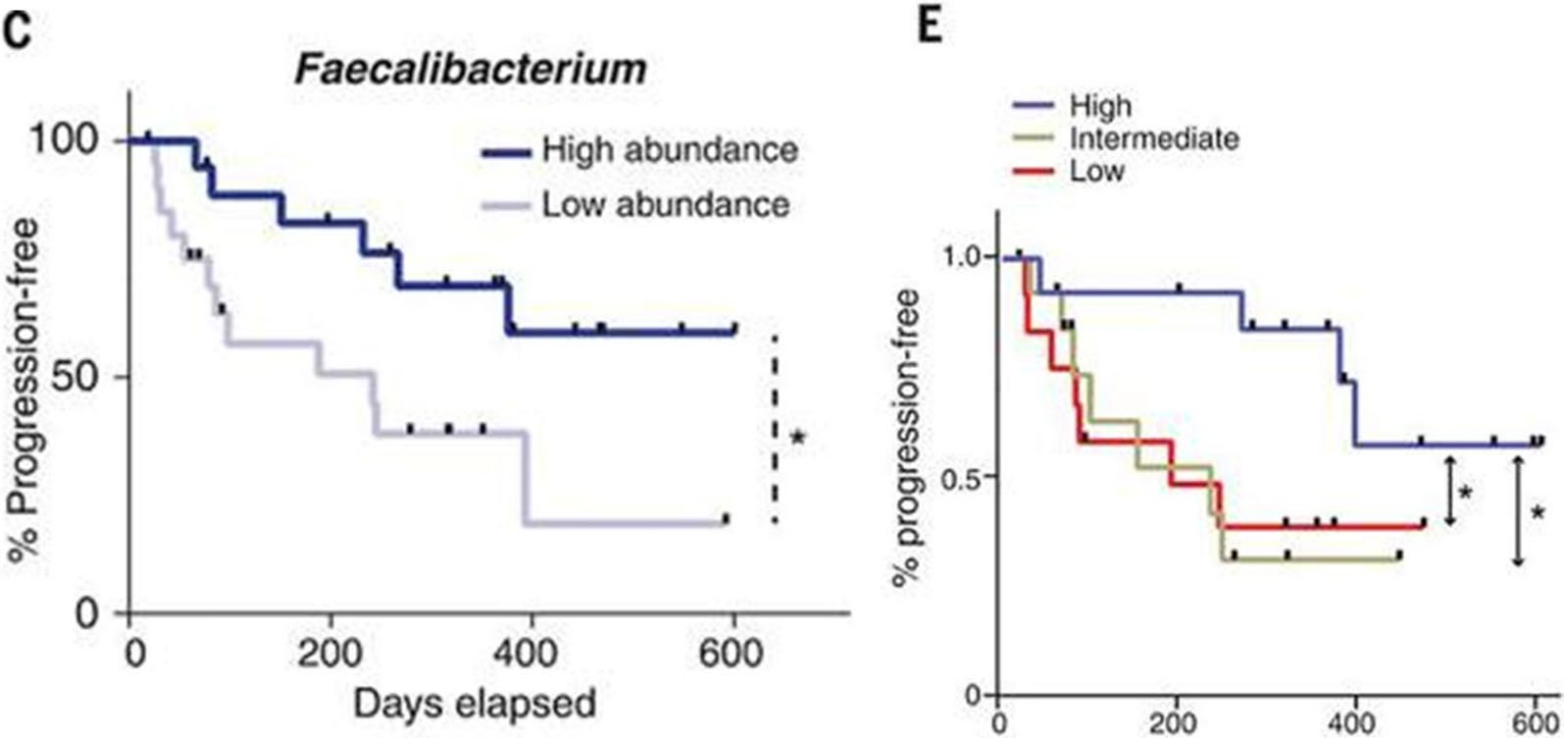
Effects of the abundance of Faecalibacterium and diversity of gut bacteria on progression-free survival in melanoma patients on PD-1 therapy. Melanoma patients on PD-1 therapy who had an increased concentration of *Faecalibacterium* (*F. prausnitzii*) in their gut microbiome experienced longer progression-free survival compared to those with low abundance. Patients with increased diversity of bacterial species in their gut microbiome also experienced longer progression-free survival [37]. Figures taken from Gopalakrishnan et al.

*F. prausnitzii* is an obligate anaerobe that normally functions to help preserve the integrity of colonic mucosa [17]. Gopalakrishnan et al. demonstrated that patients with melanoma who had an increased concentration of *F. prausnitzii* in their microbiome experienced longer progression-free survival compared to those with low abundance of *F. prausnitzii* (Fig. 4) [37]. This increase in *F. prausnitzii* correlated to an increased concentration of CTLs in the tumor microenvironment leading to longer progression-free survival in these patients [37]. Chaput et al. showed that an increase in *F. prausnitzii* in the GI tracts of melanoma patients was also associated with a stronger response to anti-CTLA-4 therapy [29]. Thus, increased representation of *F. prausnitzii* in one’s microbiome could benefit melanoma patients on combination therapy with anti-CTLA-4 and anti-PD-1 therapy.

Gopalakrishnan et al. also demonstrated that melanoma patients who had a more diverse population of bacterial species in their GI tract experienced longer progression-free survival compared to those with less diverse gut microbiomes [37]. This finding suggests that the ability to respond to anti-PD-1 therapy does not rely on the presence of a single bacterial species, but rather a diverse, mixture of species of gut microbes. The effects of different bacterial species in the gut on the response to PD-1 therapy is summarized in Table 2 below.

**Table 2:** Effects of different bacterial species in the gut on response to PD-1 therapy

Bacterial species in gut	Effect on response to PD-1 therapy
*Bifidobacterium longum* + *Bifidobacterium breve*	Oral administration to melanoma-bearing mice led to decrease in tumor volume to the same degree at PD-1 therapy alone. Co-administration with PD-1 therapy led to an even greater reduction in tumor volume.
*Akkermansia muciniphila*	Increased representation in the gut leads to a more robust response to PD-1 therapy in patients with melanoma, NSCLC and RCC due to increased production of IFN-gamma producing memory T cells
*Faecalibacterium prausnitzii*	Increased representation in the gut leads to longer progression-free survival in melanoma patients via increasing the concentration of CTLs in the tumor microenvironment

## Oncomicrobiotics

### Introduction to oncomicrobiotics

Cancer ICB treatments are not only be affected by dysbiosis, but they can actually promote dysbiosis [22]. Immunotherapy can result in 2 different types of dysbiosis, detrimental dysbiosis and beneficial dysbiosis. Detrimental dysbiosis may increase the toxicity or limit the efficacy of cancer treatments while beneficial dysbiosis may lead to increased clinical efficacy or may even be required for efficacy [13]. The ability of ICB treatments to alter the microbiome suggests that altering one’s gut microbiome prior to ICB treatment may be used as an adjuvant to cancer treatment.

The recent discoveries that alterations of gut microbes improve the efficacy of ICB therapy has led to the idea of “oncomicrobiotics”. Oncomicrobiotics are drugs, compounds or microbes that are used to selectively manipulate one’s microbiome to optimally respond to ICBs with minimal IRAEs. Daillere et al. describes *Enterococcus hirae* (*E. hirae*) and *Barnesiella intestinohominis* (*B. intestinohominis*) as oncomicrobiotics that augment the efficacy of cyclophosphamide in the treatment of lung cancer and ovarian cancer. *E. hirae* leads to an increased ratio of CD8:Treg cells in the tumor microenvironment while B. intestinohominis leads to an increase in IFN-gamma producing T cells in intratumoral lesions [38]. Both lead to a longer progression-free survival in lung and ovarian cancer due to a robust, species-specific memory Th1 cell response [38]. The effects of *E. hirae* and *B. intestinohominis* shows promise in the development of oncomicrobiotics for the treatment of other types of cancer.

It has been very difficult to develop such oncomicrobiotics due to the extensive inter-individual variation in the composition of GI bacteria [13]. In addition, establishing and maintaining exogenous bacterial species into the human GI tract is extremely difficult and variable [4]. However, gene products from gut microbes are being cataloged to generate databases that will be used to develop these oncomicrobiotics [4]. Although oncomicrobiotics are far from FDA approval, there are 4 potential ways that one could alter the microbiome: antibiotics, probiotics, postbiotics and prebiotics.

### Antibiotics in the development of oncomicrobiotics

The first and most obvious choice for the development of oncomicrobiotics is antibiotics. It may be possible to develop antibiotics that selectively kill bacteria associated with immunosuppression or IRAEs [4]. Vancomycin has shown to increase anti-CTLA-4 efficacy by selectively killing gram-positive bacteria and retaining gram-negative species such as Bacteroidales and Burkholderiales [1, 22]. However, most common antibiotics have limited selectivity and thus, target beneficial microbes as well. This makes it difficult for such antibiotics to promote beneficial dysbiosis. The development of more specific antibiotics is key to selectively manipulating one’s gut microbiota. Also, the gut microbiome is very sensitive to antibiotics and many chemotherapeutic drugs rely on gut microbiota for biotransformation and subsequent clinical response [39]. Thus, ideal antibiotics used as oncomicrobiotics would need to be extremely selective to promote beneficial dysbiosis without disrupting drug metabolism or allowing pathogenic bacteria to proliferate. Creation of such highly selective antibiotics that do not affect drug metabolism or promote detrimental dysbiosis will be a very challenging task for future researchers.

### Probiotics in the development of oncomicrobiotics

A second option for the development oncomicrobiotics is probiotics. These probiotics would be living, immunogenic commensal bacteria that are involved in anti-tumor immune responses, such as Bifidobacterium for anti-PD1 therapy [35]. Although probiotics have shown promise in helping treat some cancers, many studies using mice and other animals do not show identical and reproducible results in human studies [1]. In addition, many species of bacteria introduced to the gut are not maintained but transiently pass through the GI tract [9]. Thus, even if such commensal bacteria could be identified and introduced to the GI system via probiotics, there is no guarantee these species will be maintained in the gut and could be lost in feces. Finally, many probiotics are not regulated by the FDA and may significantly vary in quality, composition and authenticity [40].

### Postbiotics in the development of oncomicrobiotics

It may be possible to successfully introduce derivatives from commensal microbes into the GI tract. These derivatives could stimulate the immune system in a similar manner as the microbes themselves. These non-viable microbial products that are able to elicit biological responses in their host are known as “postbiotics” [13]. Postbiotics would not need to be maintained in the gut and could possibly overcome the limitation of probiotics. More research into the potential benefits of postbiotics is needed to test this hypothesis.

### Prebiotics in the development of oncomicrobiotics

The fourth candidate for successful oncomicrobiotics is prebiotics. Prebiotics are non-digestible food ingredients that stimulate the growth or activity of specific bacteria in the gut [9]. Prebiotics must be indigestible in order to exert their effects or else the body may inactivate them. Prebiotics may improve functions of certain bacteria rather than promoting growth [13]. However, just like probiotics, prebiotics may be excreted in feces prior to stimulating beneficial microbes.

### Ideal oncomicrobiotics

Regardless of which mechanism an oncomicrobiotic uses, the goal is to tailor one’s gut microbiota to maximally respond to ICB therapy. For maximum CTLA-4 blockade efficacy, the use of oncomicrobiotics that increase the representation of *B. fragilis*, *B. cepacia*, *B. thetaiotaomicron* and the *Faecalibacterium genus* in the gut would be the most beneficial. It would also be ideal to increase bacteria involved in polyamide transport and vitamin B synthesis in the gut to have maximum resistance to colitis. For maximum PD-1 blockade efficacy, the use of oncomicrobiotics that increase the representation of *Bifidobacterium breve* and *longum* in the gut would be favored. In fact, Sivan et al. demonstrated that a probiotic cocktail of *B. breve* and *B. longum* augmented the anti-tumor effects of PD-1 therapy [35]. Ideal oncomicrobiotics used to augment PD-1 therapy would also increase the abundance of *A. mucinophila* and *F. prausnitzii* in patient’s GI tract.

As the researchers continue to unfold the relationship of gut microbes and immunotherapeutic cancer treatments, more attention should be placed on the development of oncomicrobiotics. It is likely that a combination of antibiotics, probiotics, postbiotics and prebiotics will need to be isolated to allow the proper composition of gut microbiota for maximum ICB therapy efficacy and limited toxicity. However, the ability to establish and maintain these bacterial species in the human GI tract without disrupting beneficial gut bacteria will be a significant challenge in the creation of these drugs.

## Conclusions

There are many factors that are involved in the efficacy and toxicity of ICB cancer treatments. Among these factors, it seems that gut microbiota play an integral role.

Our understanding of the complex relationship between immunotherapy and the gut microbiome has come a long way in the past decade, but it is just the tip of the iceberg. Several species of bacteria have been identified to be beneficial in responding to ICB treatments, but it is likely that many other species of bacteria play a significant role. Identification of these bacteria and subsequent incorporation into the microbiome will help patients in their fight against cancer. A possible way to achieve increased efficacy and decreased toxicity of ICB treatments lies in the development of oncomicrobiotics. However, the creation of these oncomicrobiotics is a daunting task. As researchers begin to further understand the complex symbiosis between humans and bacteria, perhaps the widespread use of oncomicrobiotics will help cancer patients experience prolonged survival and a better quality of life.

## Data Availability

All data analyzed during the current study can be found in the published article.

## Abbreviations

GI: Gastrointestinal
GF: Germ-free
APC: Antigen presenting cell
MHC: Major histocompatibility complex
ICB: Immune checkpoint blockade
CTLA-4: Cytotoxic T lymphocyte antigen 4
PD-1: Programmed death 1
IRAE: Immune related adverse event
Treg: T regulatory cell
CTL: Cytotoxic T lymphocyte
